# Antibody–Drug Conjugates in Breast Cancer: Navigating Innovations, Overcoming Resistance, and Shaping Future Therapies

**DOI:** 10.3390/biomedicines13092227

**Published:** 2025-09-10

**Authors:** Hussein Sabit, Salma Abbas, Moataz T. El-Safoury, Engy M. Madkour, Sahar Mahmoud, Shaimaa Abdel-Ghany, Yasser Albrahim, Ibtesam S. Al-Dhuayan, Sanaa Rashwan, Ahmed El-Hashash, Borros Arneth

**Affiliations:** 1 Department of Medical Biotechnology, College of Biotechnology, Misr University for Science and Technology, P.O. Box 77, Giza 3237101, Egypt; 2 Department of Agri-Biotechnology, College of Biotechnology, Misr University for Science and Technology, P.O. Box 77, Giza 3237101, Egypt; 3 Department of Bioinformatics and Functional Genomics, College of Biotechnology, Misr University for Science and Technology, P.O. Box 77, Giza 3237101, Egypt; 4 Department of Pharmaceutical Biotechnology, College of Biotechnology, Misr University for Science and Technology, P.O. Box 77, Giza 3237101, Egypt; engi.mohdy@must.edu.eg; 5 Institut de Génétique Humaine, Université de Montpellier, CNRS, 34094 Montpellier, France; sahar.tarek@must.edu.eg; 6 Department of Environmental Biotechnology, College of Biotechnology, Misr University for Science and Technology, P.O. Box 77, Giza 3237101, Egypt; 7 Ministry of Health, Alahsa 39182, Saudi Arabia; 8 Department of Biology, College of Science, Imam Abdulrahman Bin Faisal University, P.O. Box 1982, Dammam 31441, Saudi Arabia; 9 University Hospital of Leicester NHS Trust, Leicester LE5 4PW, UK; 10 Elizabeth City State University Campus of the University of North Carolina, Elizabeth City, NC 27909, USA; 11 Institute of Laboratory Medicine and Pathobiochemistry, Molecular Diagnostics, Hospital of the Universities of Giessen and Marburg (UKGM), Philipps University Marburg, Baldinger Str, 35042 Marburg, Germany; 12 Institute of Laboratory Medicine and Pathobiochemistry, Molecular Diagnostics, Hospital of the Universities of Giessen and Marburg (UKGM), Justus Liebig University Giessen, 35392 Giessen, Germany

**Keywords:** breast cancer, antibody–drug conjugates, ADCs, targeted therapy, drug resistance, AI

## Abstract

Antibody–drug conjugates (ADCs) have revolutionized breast cancer (BC) therapy by combining targeted antibody specificity with potent cytotoxic payloads, thereby enhancing efficacy while minimizing systemic toxicity. This review highlights significant innovations driving ADC development alongside persistent challenges. Recent advancements include novel antibody–drug conjugate (ADC) designs targeting diverse antigens, such as HER2, HER3, and CD276, demonstrating potent anti-tumor activity and improved strategies for drug delivery. For instance, dual-payload ADCs and those leveraging extracellular vesicles offer new dimensions in precision oncology. The integration of ADCs into sequential therapy, such as sacituzumab govitecan with TOP1/PARP inhibitors, further underscores their synergistic potential. Despite these innovations, critical challenges remain, including tumor heterogeneity and acquired drug resistance, which often involve complex molecular alterations. Moreover, optimizing ADC components, including linker chemistry and payload characteristics, is essential for ensuring stability and minimizing off-target toxicity. The burgeoning role of artificial intelligence and machine learning is pivotal in accelerating the design of ADCs, target identification, and personalized patient stratification. This review aims to comprehensively explore the cutting-edge innovations and inherent challenges in ADC development for BC, providing a holistic perspective on their current impact and future trajectory.

## 1. Introduction

Antibody–drug conjugates (ADCs) represent a transformative class of therapeutics that have significantly advanced the treatment landscape for breast cancer (BC), offering a potent combination of chemotherapy’s cytotoxic power and the precise targeting capabilities of monoclonal antibodies [1,2]. These sophisticated biological constructs are designed to selectively deliver highly potent cytotoxic payloads directly to cancer cells, thereby maximizing therapeutic efficacy while minimizing systemic toxicity to healthy tissues [2]. The emergence of ADCs has provided new avenues for addressing complex challenges in BC, particularly in patient populations with limited treatment options or those who have developed resistance to conventional therapies [3,4]. The intricate design of ADCs, comprising an antibody, a stable linker, and a cytotoxic drug, underpins their mechanism of action, which involves specific antigen recognition, receptor-mediated internalization, and intracellular payload release [2]. This targeted delivery system holds immense promise for improving patient outcomes by enhancing anti-tumor activity and mitigating off-target side effects, which are common limitations of traditional chemotherapy.

Recent innovations in ADC development are continually pushing the boundaries of their therapeutic potential, addressing critical challenges such as tumor heterogeneity, drug resistance, and the need for personalized treatment approaches. For instance, the concept of sequential therapy, where ADCs are integrated into treatment strategies, has shown promise in breast cancer (BC). Sacituzumab govitecan, an ADC, has enabled a sequential TOP1/PARP-inhibitor therapy strategy in BC patients, highlighting synergistic therapeutic possibilities [5]. Furthermore, novel ADC designs are emerging to combat resistance mechanisms, including those that incorporate dual payloads or target multiple antigens to improve efficacy in diverse tumor landscapes [6,7]. The development of a novel bispecific ADC targeting HER2 and HER3, for example, has demonstrated potent therapeutic efficacy against BC, indicating a promising strategy for overcoming limitations associated with single-target approaches [7]. Beyond conventional ADCs, advancements in drug delivery systems are also contributing to the field, with antibody-displaying extracellular vesicles being explored for targeted cancer therapy, offering a new dimension to precision medicine [8].

Despite these significant strides, several challenges persist in the broader application and optimization of ADCs for BC. Tumor heterogeneity, a hallmark of breast cancer (BC), poses a substantial hurdle, as variable antigen expression across tumor cells can limit the effectiveness of ADCs and contribute to drug resistance [4]. The emergence of acquired resistance to ADCs, often involving alterations in DNA repair pathways or other molecular mechanisms, necessitates ongoing research into novel therapeutic strategies [9,10]. Efforts are underway to address these resistance mechanisms, with some research focusing on the DNA repair pathway as a therapeutic target to synergize with ADCs, such as trastuzumab deruxtecan, in resistant HER2-overexpressing breast cancer [11]. The optimization of ADC components, including the linker chemistry and payload characteristics, remains critical to enhance drug stability in circulation and ensure efficient, selective release at the tumor site while minimizing systemic toxicity [12,13].

The landscape of ADC development is also expanding to include novel targets beyond HER2, such as CEACAM5, Claudin-2, and DDR1, broadening the applicability of ADCs to various BC subtypes [13,14,15]. For instance, a high-affinity ADC targeting CEACAM5-positive tumors has shown potent and selective cytotoxicity, indicating its potential in clinical oncology [13]. The role of artificial intelligence (AI) and machine learning (ML) is rapidly expanding, offering sophisticated tools for identifying novel targets, optimizing ADC design parameters, and predicting drug response, thereby accelerating the drug discovery and development pipeline [16]. Furthermore, the concept of personalized ADC therapy, leveraging advanced biomarker identification and quantitative systems pharmacology (QSP) models, is central to tailoring treatments to individual patient profiles, thereby maximizing efficacy and minimizing adverse events [16,17]. This comprehensive review will delve into the innovations driving ADC development for BC, critically examine the persistent challenges, and explore future directions poised to enhance their therapeutic impact.

## 2. Innovations in ADC Development for BC

### 2.1. Evolving Antibody Targets in BC ADCs

Historically, human epidermal growth factor receptor 2 (HER2) has been the most extensively investigated target for the development of antibody–drug conjugates (ADCs) in breast cancer (BC). Trastuzumab emtansine (T-DM1), the first HER2-targeted ADC approved for metastatic HER2-positive BC, consists of an anti-HER2 antibody linked via a non-cleavable thioether to the tubulin polymerization inhibitor, DM1, which induces cell death [18]. Despite its therapeutic benefits, both innate and acquired resistance, often due to impaired drug internalization and reduced or heterogeneous HER2 expression, pose significant challenges to sustained clinical response [19,20].

Strategies to circumvent or delay T-DM1 resistance include combination therapies with other drugs [21,22] as well as the development of next-generation anti-HER2 ADCs with improved designs. Trastuzumab deruxtecan (T-DXd), an anti-HER2 monoclonal antibody conjugated to a topoisomerase I inhibitor via a tetrapeptide-based cleavable linker, has gained approval for metastatic HER2-positive BC [23]. This ADC demonstrates superior efficacy over other HER2-targeting ADCs, effectively targeting tumor cells with low HER2 expression and exhibiting bystander effects [24,25,26]. Furthermore, several other novel HER2-targeted ADCs, such as ARX788, RC48-ADC, and T-VEd9, are currently undergoing clinical investigation and show promise as emerging candidates for next-generation HER2-targeted therapies [27,28,29].

Beyond HER2, a limited number of studies have explored ADCs targeting epidermal growth factor receptor 1 (EGFR/HER1) or 3 (HER3) for BC therapy. The ADC LR004-VC-MMAE demonstrated potent antitumor activity by suppressing EGFR signaling and downregulating cancer stemness-associated genes, suggesting its potential as a therapeutic candidate for EGFR-positive triple-negative BC [30]. A more recent strategy involves an advanced ADC that targets EGFR on the tumor cell surface while delivering a CDK inhibitor to disrupt dysregulated cell cycle signaling, offering a potential treatment option for patients with EGFR-expressing, therapy-resistant triple-negative breast cancer (TNBC) [31].

HER3-DXd, an ADC targeting HER3, has demonstrated meaningful clinical activity across various BC subtypes, including heavily pretreated patients and those with diverse HER3 expression levels. Despite associated toxicities, its efficacy, particularly in difficult-to-treat populations like triple-negative and HER2-positive BCs, underscores its therapeutic potential [32]. Further studies have affirmed the acceptable safety profile of HER3-DXd and validated its promise as a therapeutic option for patients with HER3-expressing metastatic BC [33].

Another promising target, Trophoblast cell surface antigen 2 (TROP2), is being explored for the development of ADCs in BC. Sacituzumab govitecan (SG), which consists of anti-TROP2 monoclonal antibodies linked to a topoisomerase I inhibitor, received accelerated FDA approval in April 2020 for metastatic TNBC [34]. Subsequent studies have validated its clinically meaningful antitumor activity and manageable safety profile [35,36]. Primary results also support SG as an effective treatment option for patients with endocrine-resistant HR+/HER2− metastatic BC, improving quality of life with manageable safety in a heavily pretreated population [37,38]. Ongoing clinical trials are further investigating the effectiveness of datopotamab deruxtecan (Dato-DXd) for HR+/HER2− metastatic BC patients [39]. Additionally, sacituzumab tirocentin, a novel TROP2-directed ADC with a new linker, demonstrated substantial clinical benefit over chemotherapy in advanced TNBC, significantly improving progression-free and overall survival and exhibiting a higher objective response rate with manageable toxicity [40]. Building on TROP2-targeted ADC advancements, Dato-DXd has demonstrated potent antitumor activity and efficient payload delivery in TROP2-expressing preclinical models, accompanied by a favorable safety profile, which supports its therapeutic potential [41].

### 2.2. Advancements in Linker Technology

The linker, which connects the antibody to its cytotoxic payload in an ADC, is crucial for determining compound stability; drug release kinetics; and, ultimately, therapeutic efficacy and safety.

Cleavable linkers are designed to exploit the distinct microenvironments of tumor cells, enabling precise intracellular release of cytotoxic agents. These include chemically cleavable types (e.g., hydrazone and disulfide bonds, which are responsive to pH or redox conditions) and enzyme-sensitive linkers (e.g., glucuronide and peptide bonds, which are cleaved by tumor-specific enzymes) [42].

In contrast, non-cleavable linkers, such as thioether or maleimidocaproyl types, remain intact under physiological conditions, releasing the payload only after the entire ADC is internalized and cellular proteases degrade the antibody. While cleavable linkers enable selective drug release, non-cleavable linkers often provide enhanced plasma stability, potentially minimizing off-target toxicity from premature drug liberation [42].

Conventional peptide-cleavable linkers face limitations, including hydrophobicity-induced aggregation, low drug-to-antibody ratios (DARs), and susceptibility to premature enzymatic cleavage. Novel strategies to overcome these challenges include a new cleavable linker drug platform that improves ADC stability and efficacy through site-specific conjugation via farnesyl transferase and optimized β-glucuronide chemistry for enhanced circulation stability and intracellular payload release [43]. Another innovative approach, an exolinker system, places the cleavable peptide at the exo position and incorporates hydrophilic elements. This design enhances stability, enables higher DARs, minimizes aggregation, and demonstrates strong resistance to enzymatic degradation, supporting its potential for safer and more effective ADCs [44].

### 2.3. Innovative ADC Formats and Conjugation Strategies

Site-specific antibody conjugation is a powerful strategy for improving ADCs by controlling drug-to-antibody ratios (DARs), homogeneity, stability, and therapeutic index. Several protocols have emerged to enhance site selectivity without requiring extensive antibody engineering.

One approach involves creating specific “docking points” on antibodies by introducing single or double unpaired cysteines at selected sites in the antibody’s constant heavy chain domains (CH1, CH2, and CH3). These engineered sites enable precise, efficient attachment of drugs with minimal off-target conjugation, resulting in ADCs with consistent and well-controlled drug loading [45]. Another method employed a thioester-based acyl transfer reagent to directly attach payloads to lysine 251 of native IgGs in a single step, yielding homogeneous, stable, and active ADCs [46]. Similarly, the AJICAP platform utilizes Fc-binding peptides to site-selectively modify lysines 248 or 288 on native antibodies without requiring redox treatment, enabling the generation of over 20 ADCs with minimal aggregation [47].

Building on these advances, a novel ADC format for triple-negative BC utilizes single-chain variable fragments (scFvs) targeting EGFR or the epithelial cell adhesion molecule (EpCAM) instead of full-length antibodies. These scFvs are fused to a SNAP tag, facilitating rapid and precise conjugation to benzylguanine (BG)-modified monomethyl auristatin E (MMAE). This design achieves potent antigen-specific cytotoxicity in vitro while offering improved tumor penetration and greater flexibility in ADC construction, representing a promising therapeutic approach for TNBC [48].

These studies collectively demonstrate that innovative site-specific conjugation techniques, including engineered cysteines, thioester chemistry, Fc-binding peptides, and SNAP-tag technology, enable the creation of more homogeneous, stable, and effective ADCs, thereby expanding therapeutic options across diverse cancer types.

Recent advancements also address the complexities of incorporating dual payloads, which traditionally involved branched linkers or multi-step processes that could compromise efficiency or increase hydrophobicity. A streamlined one-pot method has been developed to generate dual-site ADCs with defined degrees of amino acid replacement (DARs) at both the N-glycosylation and K248 sites. This approach enables the attachment of either identical or distinct payloads, resulting in highly homogeneous, stable, and enhanced-performance ADCs in both in vitro and in vivo models [49].

### 2.4. Next-Generation Cytotoxic Payloads

**Figure 1:**
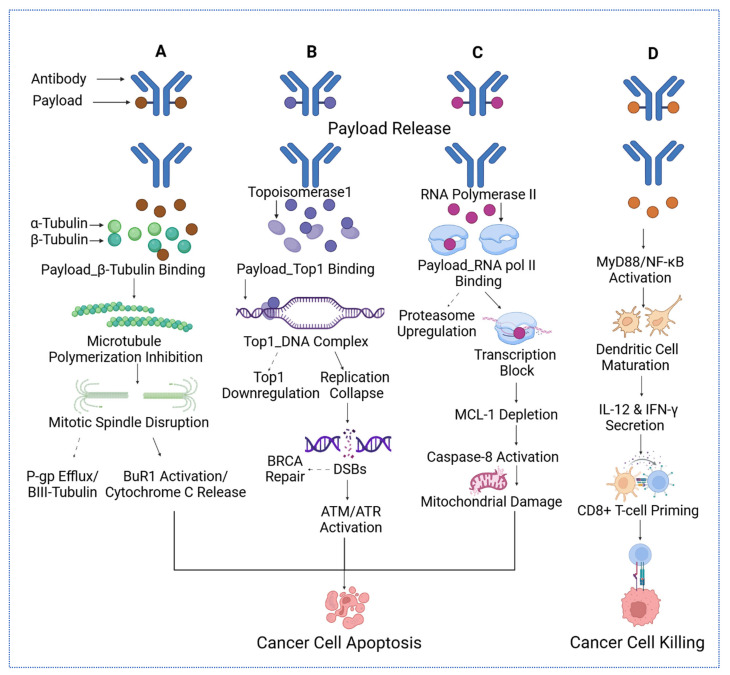
The four distinct mechanisms of cytotoxic payloads delivered via antibodies to target cancer cells. (**A**) Microtubule inhibitors bind α- and β-tubulin, disrupting mitotic spindle formation, inhibiting polymerization, and triggering cytochrome C release and apoptosis, while countering P-gp efflux and activating BuR1. (**B**) DNA-damaging payloads, such as topoisomerase inhibitors, form Top1–DNA complexes, downregulate replication, deplete MCL-1, and induce mitochondrial damage and caspase-8 activation, leading to apoptosis via ATM/ATR and BRCA-DSB repair pathways. (**C**) Transcription disruptors targeting RNA polymerase II block transcription, upregulate proteasomes, and activate caspase-8, causing mitochondrial damage and cancer cell death. (**D**) Immune modulators activate MyD88/NF-κB, promoting dendritic cell maturation, IL-12 and IFN-γ secretion, and CD8+ T-cell priming, resulting in effective cancer cell killing. For the arrow, the straight arrows reflect the mechanisms that eventually lead to cancer cell apoptosis, while the dashed arrows reflect the mechanisms that might reverse the cancer cell apoptosis.

Beyond conventional microtubule-disrupting payloads, such as auristatins and maytansinoids, topoisomerase I inhibitors have emerged as promising alternatives. HER3-DXd, an ADC comprising a fully human anti-HER3 monoclonal antibody (patritumab) linked to a topoisomerase I inhibitor, has demonstrated a manageable safety profile and clinical activity across varying levels of HER3 expression [33] (Figure 1).

Building on this class of payloads, trastuzumab-L6, a HER2-targeted ADC with a novel topoisomerase I inhibitor (MF-6), was designed to trigger immunogenic cell death and stimulate antitumor immunity. In preclinical models, it activated dendritic cells and cytotoxic T lymphocytes, inducing long-term immune memory and positioning trastuzumab-L6 as both a cytotoxic and an immunostimulatory ADC [50].

In addition to topoisomerase inhibitors, ADCs now incorporate novel payloads, including cyclin-dependent kinase (CDK) inhibitors and immune modulators. A cetuximab-based ADC delivering the CDK inhibitor SNS-032 selectively targets EGFR-expressing TNBC cells, overcoming resistance linked to CDK2 activity and demonstrating strong antitumor effects with minimal toxicity [14]. Similarly, a HER2-targeted ADC carrying a Toll-like receptor 7 (TLR7) agonist activates innate immune responses within tumors, inducing tumor regression through immune stimulation rather than direct cytotoxicity [51]. Collectively, these advances highlight the expanding range of ADC payloads that not only improve efficacy and overcome resistance but also offer enhanced solubility, reduced systemic toxicity, and novel immunomodulatory properties.

### 2.5. Multi-Modal Approaches and Combination Therapies

Combining ADCs with immunotherapy shows promising results, particularly in TNBC [52]. In first-line advanced or metastatic TNBC, Dato-DXd combined with durvalumab (an immune checkpoint inhibitor targeting PD-L1) achieved a 74% response rate with manageable toxicity. This synergy arises from the ADC’s direct tumor-killing effect and durvalumab’s immune activation [53]. Similarly, a phase II trial evaluates avelumab (another immune checkpoint inhibitor) combined with either SG or liposomal doxorubicin to enhance immune response and tumor targeting [54]. These studies underscore the growing potential of ADC-immunotherapy combinations in expanding effective treatment strategies for TNBC.

Clinical trials are also evaluating the safety and potential synergy of combining ADCs with inhibitors of DNA damage response. For example, sacituzumab govitecan, the ADC that delivers a topoisomerase I inhibitor to tumor cells, is being combined with talazoparib, a poly ADP-ribose polymerase (PARP) inhibitor. This combination aims to enhance tumor cell killing by delivering targeted cytotoxic therapy while simultaneously preventing cancer cells from repairing DNA damage [55].

The combination of ADCs and chemotherapy is also emerging as a promising strategy in TNBC. Preclinical studies demonstrated synergistic effects when SG was combined with platinum-based agents such as carboplatin or cisplatin in multiple tumor models, including TNBC. These combinations promoted pro-apoptotic signaling, disrupted cell cycle progression, and achieved significant in vivo antitumor activity with good tolerability [56,57].

Furthermore, dual-payload ADCs offer a novel and powerful approach to addressing tumor heterogeneity and resistance in BC. A HER2-targeted ADC carrying two cytotoxic agents demonstrated superior efficacy and survival benefits compared to single-payload ADCs in resistant, heterogeneous tumor models, with minimal toxicity and favorable pharmacokinetics [58]. Another study developed a dual ADC targeting CD276 (B7-H3), which is highly expressed in TNBC, combining a cytotoxic drug with a Toll-like receptor agonist. This approach achieved up to 100% tumor reduction in animal models and enhanced immune activation in the tumor microenvironment. These findings underscore the potential of dual-payload ADCs to improve outcomes in aggressive, treatment-resistant BCs [6].

## 3. Challenges in ADC Development and Application for BC

### 3.1. Tumor Heterogeneity and Drug Resistance

Tumor heterogeneity, both within the primary tumor and across metastatic sites, significantly limits the effectiveness of ADCs in BC treatment. A comprehensive study of metastatic BC patients revealed considerable variability in HER2 expression among different tumor sites, raising concerns about relying on single biopsies or current HER2-low classifications for guiding ADC therapy decisions [59]. Moreover, a clinical trial for early-stage HER2-positive BC observed that tumors with heterogeneous HER2 expression showed no complete response to HER2-targeted therapy with trastuzumab emtansine and pertuzumab, whereas tumors with homogeneous HER2 expression achieved a 55% complete response rate [20]. This highlights that even small populations of HER2-negative cells within a tumor can confer resistance to ADC treatment.

Resistance mechanisms to ADCs can also arise from alterations in the drug clearance pathway or changes in payload-specific targets. For instance, increased expression of the ATP-binding cassette transporter ABCB1 has been observed in cell lines resistant to brentuximab vedotin, resulting in greater efflux of the cytotoxic drug monomethyl auristatin E (MMAE) [60]. A similar pattern of elevated ABCB1 levels was observed in tumors resistant to the anti-nectin-4 ADC enfortumab vedotin [61]. Furthermore, resistance to ADCs carrying topoisomerase inhibitors may be caused by alterations in topoisomerase expression or downstream signaling pathways [62].

To address these challenges, novel ADC designs are being developed. One strategy involves creating ADCs that simultaneously bind to two distinct receptors, such as receptor d’origine nantais and mesenchymal-epithelial transition factor. This dual-targeting ADC effectively overcame the limitations of single-target therapies, enhancing drug distribution and therapeutic response in heterogeneous cancers [63]. Another approach to target tumor heterogeneity involves conjugating a single antibody with two distinct cytotoxic medicines, which demonstrated superior tumor killing and survival benefits compared to combinations of single-drug ADCs in models of HER2 heterogeneity and resistance [58].

### 3.2. ADC Toxicity and Component Impact

The toxicity profiles of FDA-approved ADCs and the contributions of their constituent parts have been comprehensively assessed. While DNA-damaging drugs tended to cause more severe toxicity in hematologic malignancies, the intensity of toxicities did not differ significantly across payload classes [64]. Linker chemistry also impacts toxicity: non-cleavable linkers offer greater systemic stability but restrict drug release, whereas cleavable linkers may increase off-target effects due to premature payload release. Advanced linker designs, such as exo- and tandem-cleavable types, aim to enhance safety by allowing for regulated release within the TME [64].

Toxicities are generally driven more by the payload than by antigen expression. For example, enfortumab vedotin caused skin rash due to Nectin-4 expression in the skin, but most toxicities occurred in tissues unrelated to antigen targeting. This includes liver enzyme elevations with calicheamicin-based gemtuzumab ozogamicin, despite the absence of CD33 in the liver. Corneal toxicity has been observed with ADCs carrying a variety of payloads, including DM4, MMAF, MMAE, and duocarmycin, suggesting a vulnerability of the corneal epithelium that extends beyond the specific payload [64,65]. Although high-grade toxicities did not vary significantly across ADC classes, the data indicate that the majority of side effects are caused by the pharmacological action of the payload rather than target specificity. This highlights the need for ongoing improvements in payload and linker design to enhance safety and tolerability.

### 3.3. Patient Selection and Biomarker Development

Despite their promise, the clinical success of ADCs for BC treatment hinges on identifying reliable biomarkers to predict treatment response, monitor emerging resistance mechanisms, and optimize patient selection. LGR5 (leucine-rich repeat-containing G-protein-coupled receptor 5) has been identified as both a potential therapeutic target and a prognostic marker in ER-negative breast cancer, with anti-LGR5 ADCs showing notable effectiveness in LGR5-positive patient-derived xenograft models [66].

Beyond tissue-based methods, liquid biopsy platforms are increasingly utilized to study the dynamics of ADC resistance. For instance, circulating tumor DNA (ctDNA) studies from the DAISY trial revealed early acquisition of ESR1 and HER2 mutations associated with resistance to trastuzumab deruxtecan (T-DXd) [7]. Furthermore, comparing alternative indicators to protein expression alone, TROP2 mRNA levels may improve response prediction for TROP2-directed ADCs [67].

Advanced spatial transcriptomic techniques, such as multiplexed fluorescence in situ hybridization sequencing (mFISHseq), have been developed to overcome the limitations of traditional immunohistochemistry (IHC)-based biomarker assessment. By combining laser capture microdissection and high-resolution RNA analysis, mFISHseq offers unprecedented insight into intratumoral heterogeneity and ADC response patterns. In the I-SPY2 trial, mFISHseq analysis outperformed conventional HER2 IHC stratification by identifying a 19-gene signature predictive of T-DM1 response [68]. These results highlight how spatial transcriptomics can significantly improve patient selection beyond existing state-of-the-art techniques.

Collectively, these findings demonstrate how the integration of established and novel biomarker systems can address the intricacies of ADC therapy, from initial target selection to real-time resistance monitoring. Future research should prioritize confirming these strategies in prospective clinical studies to facilitate their incorporation into standard treatment protocols.

## 4. Antibody–Receptor Binding Kinetics and Internalization Pathways

ADCs ingeniously combine the potent cytotoxicity of chemotherapy with the targeted specificity of antibodies into a single molecule. These sophisticated molecules consist of three crucial elements: a monoclonal antibody (mAb) designed to target a specific tumor antigen, a highly potent cytotoxic payload, and a chemical linker that connects the two. The fundamental process begins with the antibody binding to its designated tumor antigen on the cell surface. Subsequently, the entire ADC is internalized and degraded within lysosomes, leading to the release of the active cytotoxic agent into the cytoplasm [20], which ultimately triggers the death of the tumor cell [69].

Monoclonal antibodies themselves typically exert their therapeutic effects through two distinct mechanisms: specific binding to a target antigen on tumor cells and the recruitment of immune cells. This immune cell recruitment can lead to the killing of target cells through antibody-dependent cellular phagocytosis (ADCP) [70], the release of cytokines via antibody-dependent cellular cytotoxicity (ADCC) [71], or complement-dependent cytotoxicity (CDC) [72]. Building on the inherent selectivity of mAbs and the formidable power of small-molecule cytotoxins, ADCs have been developed further to enhance the selectivity and efficacy of targeted therapy [73].

For an optimal ADC design, selecting an antibody with high affinity and specificity for a new antigen is paramount. Moreover, this target antigen should predominantly be expressed on the surface of the target cell, with only trace amounts present in healthy cells. To facilitate the intracellular transport and release of the cytotoxic payload, the target antigen should also internalize rapidly. The ADC itself typically features a humanized monoclonal antibody, chosen for its extended circulatory half-life and reduced immunogenicity. This antibody is conjugated to a powerful cytotoxic agent, designed for minimal off-target effects, via a linker that remains stable in circulation to prevent premature release yet is cleavable to facilitate intracellular payload release or release within the TME [42,74].

Upon administration, the antibody component of the ADC first attaches to the tumor-specific antigen. Following this binding, the entire ADC–antigen complex undergoes receptor-mediated endocytosis [75,76]. This process of binding to specific epitopes, as seen with antibodies against EGFR, can accelerate internalization [77]. Once internalized, the antigen–ADC complex can either be recycled back to the cell surface or directed along the lysosomal pathway for intracellular drug release and enzymatic degradation. The cell’s internalization capacity plays a significant role in determining the amount of ADC that can accumulate intracellularly [77].

### 4.1. Intracellular Trafficking and Lysosomal/Endosomal Processing

While pathways can vary considerably depending on the specific antigen and ADC design, many studies support a general understanding of ADC internalization and trafficking in cancer cells. The overall procedure involves antigen identification on the tumor cell surface; followed by receptor-mediated endocytosis; intracellular trafficking through endosomal compartments; and, ultimately, payload release in the lysosome [69,78].

Antigen Recognition and Binding: ADCs are specifically engineered to target tumor-associated antigens that are overexpressed on the surface of cancer cells. The monoclonal antibody (mAb) component of the ADC binds with high selectivity to these antigens, a critical step for subsequent ADC internalization and its therapeutic effectiveness.

Receptor-Mediated Endocytosis: After binding, the ADC–antigen complex is internalized into the cell through receptor-mediated endocytosis. This process can occur via several distinct mechanisms. Clathrin-mediated endocytosis is a primary pathway where the ligand–receptor complex clusters into clathrin-coated pits, invaginates, and forms vesicles that detach from the membrane. Caveolae-mediated endocytosis involves the internalization of the ADC through flask-shaped invaginations in the plasma membrane rich in caveolin proteins. Macropinocytosis, a type of endocytosis, entails the absorption of extracellular fluid and solutes into large vesicles. Although less specific, this mechanism may also contribute to ADC uptake. The efficiency and precise pathway of internalization can vary based on the specific antigen and the ADC’s design.

Endosomal Maturation and Lysosomal Fusion: The absorbed ADC is subsequently transported from early endosomes to late endosomes, where it eventually merges with lysosomes. Lysosomes, acidic organelles containing hydrolytic enzymes such as cathepsins, are essential for the breakdown of ADC. In this acidic environment, the linker connecting the antibody and the cytotoxic payload must be cleaved.

Payload Release and Mechanism of Action: Following intracellular cleavage, the cytotoxic payload is liberated into the cytoplasm, where its effect is exerted based on its specific molecular target, as detailed in Table 1. The cytotoxic agent typically blocks critical cellular mechanisms necessary for cancer survival, such as DNA or microtubule assembly, thereby leading to the apoptosis of tumor cells.

FcRn-Mediated Recycling: Within endosomes, certain ADCs may interact with the neonatal Fc receptor (FcRn). This interaction can result in the ADC being recycled back to the cell surface, potentially reducing the amount of medication released into the cell. Therefore, methods for designing ADCs that do not bind to FcRn are currently being investigated to enhance intracellular drug delivery.

### 4.2. The Bystander Effect

A key characteristic of certain ADCs is the bystander effect, which significantly enhances therapeutic efficacy, particularly in tumors with heterogeneous antigen expression. This phenomenon occurs when the released cytotoxic payload, particularly if it is membrane-permeable (like MMAE or DXd), diffuses from the target antigen-positive cell into adjacent antigen-negative tumor cells, leading to their subsequent death. This capability is particularly relevant in solid tumors, where the non-uniform expression of target antigens often limits the overall effectiveness of treatment. A recent study [79] demonstrated that HER2-targeted ADCs exhibit measurable bystander activity within the tumor microenvironment. Furthermore, this effect can be amplified through coadministration with the parental unconjugated antibody, which improves tumor penetration and overcomes binding site barriers, ultimately extending the ADC’s cytotoxic reach beyond only antigen-expressing cells. Table 1 summarized the molecular mechanisms of cytotoxic payload classes in ADCs.

**Table 1:** Molecular mechanisms of cytotoxic payload classes in ADCs.

Payload Class	Representative Payload	Mechanism of Cytotoxicity	References
Microtubule Inhibitors	DM1 (in T-DM1), MMAE	Bind to tubulin, inhibit polymerization, arrest mitosis at G2/M phase, trigger apoptosis	[80]
DNA Crosslinkers/Alkylators	PBD dimer	Form DNA interstrand crosslinks, block replication/transcription, activate DNA damage response and apoptosis	[81]
DNA Cleaving Agents	Calicheamicin	Induce site-specific double-strand DNA breaks via minor groove binding and radical generation	[82]
Topoisomerase I Inhibitors	DXd (deruxtecan), SN-38	Inhibit topoisomerase I, stabilize DNA cleavable complexes, result in replication stress and double-strand breaks	[41]
RNA Polymerase II Inhibitors	α-Amanitin	Binds the bridge helix of RNA polymerase II that inhibits transcription, leading to apoptosis in highly active cells	[83]
BCL-XL Inhibitor Conjugates	BCL-XL toxin derivatives (experimental)	Promote apoptosis by inhibiting BCL-XL, disrupting mitochondrial membrane potential, enhancing chemosensitivity	[84]

BCL-XL, B-cell lymphoma extra large; DM1, N2′-deacetyl-N2′-(3-mercapto-1-oxopropyl)-maytansine; DXd, Deruxtecan; MMAE, Monomethyl auristatin E; PBD, Pyrrolobenzodiazepine; SN-38, 7-Ethyl-10-hydroxycamptothecin.

## 5. Pharmacokinetics and Pharmacodynamics of ADCs

Understanding pharmacokinetics (PK) and pharmacodynamics (PD) is crucial for the development and clinical application of ADCs. These factors govern the drug’s movement through the body; the release of its cytotoxic payload; and, ultimately, its therapeutic and toxic effects on both cancer and healthy cells.

### 5.1. Route of Administration and Impact on PK/PD

Currently, all approved ADCs are administered intravenously (IV), with limited research exploring alternative delivery routes [85]. In contrast, monoclonal antibodies and other therapeutic proteins have demonstrated enhanced therapeutic indices when delivered via routes other than IV, including subcutaneous (SC), intramuscular, intravitreal, intra-central nervous system (CNS), inhalation, intra-articular, and intratumoral (IT) methods [86]. Among these, SC and IT routes have gained particular attention in oncology for improving the delivery of protein-based therapeutics [87,88]. Despite these advancements in protein therapies, ADC research primarily remains focused on IV administration, underscoring the need for more targeted studies to assess the feasibility and benefits of SC and IT delivery for ADCs [85].

### 5.2. Tumor Distribution and Antibody Format

The dense and tightly packed structure of tumor tissues presents a significant barrier to the effective penetration and distribution of conventional IgG-based drugs. This makes it challenging for these drugs to reach tumor cells located far from blood vessels, thereby compromising therapeutic effectiveness [89]. To overcome this challenge, researchers have explored smaller antibody fragments, such as Fab, scFv, and VH domains, which offer improved tissue penetration [90,91,92]. However, a drawback of these smaller formats is their significantly shorter half-lives compared to full-length antibodies, which leads to rapid clearance from circulation and necessitates more frequent dosing—a considerable obstacle to their clinical application.

To enhance the half-life of nanobodies and other smaller formats, several strategies have been developed, including PEGylation, fusion with the Fc domain, binding to albumin, or anti-human serum albumin (αHSA). Fc fusion, in particular, leverages the interaction between the Fc region and the neonatal Fc receptor (FcRn), a mechanism enabling pH-dependent recycling. Under the acidic conditions inside cells, Fc binds strongly to FcRn, allowing it to escape degradation and re-enter circulation. Once in the neutral pH of the bloodstream, the Fc detaches from FcRn, thereby prolonging its systemic presence [93,94]. Engineering the Fc region—for instance, to improve FcRn binding or to incorporate albumin binding—can effectively extend the half-life of ADCs by reducing their clearance rate. Conversely, unmodified small antibody fragments are typically cleared more rapidly via the kidneys due to their low molecular weight unless specific modifications are introduced to enhance their stability [9].

### 5.3. Metabolism, Catabolism, and Biotransformation

ADCs undergo various biotransformation processes within both systemic circulation and tissue cells. Key transformation pathways include linker cleavage or deconjugation, linker immolation, and metabolism of the cytotoxic payload, in addition to the typical degradation of the antibody itself. As ADCs circulate through the bloodstream and eventually release their payloads within target cells, they can produce a range of catabolites, some of which may retain pharmacological activity. Ideally, ADCs should remain stable and intact in circulation until they reach the target site; however, there are instances where breakdown products formed during biotransformation still exhibit biological activity [95,96,97]. In many cases, the metabolic stability of an ADC serves as a proxy for the concentration of the intact conjugate, with pharmacokinetic parameters such as clearance, volume of distribution, and half-life tending to remain consistent across various linker–payload combinations [98].

### 5.4. Linker Chemistry and Payload Release

Linker design critically modulates the stability of ADCs in systemic circulation and the efficiency of payload release within tumors, thereby influencing ADCs’ pharmacokinetic (PK), efficacy, and toxicity profiles. The linker, which bridges the antibody and the cytotoxic drug, plays a crucial role in the functionality of ADCs. It must ensure two essential features: robust stability during circulation and the selective release of the payload within the target tissue.

The major category is the cleavable linker, which contains a chemical trigger that enables efficient cleavage and drug release, specifically within the tumor environment. Notably, over 80% of ADCs currently approved for clinical use utilize cleavable linkers [99], exemplified by inotuzumab ozogamicin (Besponsa) and brentuximab vedotin (Adcetris) [100,101]. The other class of linkers is non-cleavable, which does not contain a chemical trigger for drug release; instead, the linker remains attached as part of the payload structure. Among the currently approved ADCs, ado-trastuzumab emtansine (Kadcyla, T-DM1) is the sole example of using a non-cleavable linker. In ADCs with non-cleavable linkers, the release of the cytotoxic agent typically occurs inside the lysosome following internalization. Within this compartment, the antibody component is broken down by proteases into peptides or amino acids, enabling the payload to be released. Notably, while payload release is often the primary rate-limiting step, lysosomal escape can also pose a barrier for some ADCs [95]. In these cases, the resulting catabolite remains biologically active, with the payload still chemically linked to a peptide or amino acid fragment. To exit the lysosome and exert its effect, this modified payload may require the assistance of a specific transporter [102]. Conversely, cleavable linkers are engineered to release the cytotoxic payload in its original, unaltered form. These linkers typically utilize chemical mechanisms that trigger a self-immolation process once the linker is cleaved, ensuring the efficient liberation of the active drug. As a result, the overall rate of payload release is influenced by both the initial cleavage of the linker and the subsequent immolation reaction [95].

### 5.5. PK/PD Variability and Biomarker Considerations

Significant variability in the expression levels of genes that may mediate sensitivity or resistance to ADCs downstream of their targets has been reported [103]. These differences in gene expression provide more detailed insight into the proportion of tumors within a specific cancer type that are likely to respond to a given ADC. To address this, researchers developed a “targetgram,” which visually represents the relative expression levels of ADC targets along with average levels of markers associated with resistance and sensitivity in individual tumor samples. This tool may prove especially valuable given the considerable patient-to-patient variability within cancer subtypes and the growing array of ADC therapies that require strategic selection and sequencing.

### 5.6. Strategies for Dose Optimization and PK/PD Modeling

ADCs present unique challenges in dose optimization due to their composite nature, combining the pharmacokinetics of both antibodies and cytotoxic agents. Advanced PK/PD modeling is increasingly employed to design dosing strategies that maximize efficacy while minimizing toxicity. For instance, a mechanistic model incorporating tumor penetration and growth inhibition dynamics has been developed, revealing that dosing frequency and payload release kinetics significantly influence intratumoral drug concentration and therapeutic outcomes [104]. These insights support model-based decision-making for selecting dose levels and schedules that improve target tissue exposure while reducing systemic toxicity. Additionally, strategies such as fixed dosing, body weight cap dosing, and treatment duration limitations—applied in approved ADCs—have been highlighted to balance interpatient variability and improve the therapeutic index [105]. Model-informed drug development (MIDD) approaches, grounded in quantitative systems pharmacology, have become essential tools for ADC design and clinical translation.

## 6. Preclinical Evaluation and Translational Challenges

To advance ADCs from the laboratory to clinical use for BC patients, researchers heavily rely on preclinical models that accurately simulate tumor behavior. These models serve as critical proving grounds for establishing the safety and effectiveness of new therapies [28].

### 6.1. Preclinical Models for ADC Evaluation

In vitro models, including cell lines and three-dimensional (3D) cultures, play a pivotal role in the initial evaluation of ADCs. These models are instrumental in understanding the interaction of ADCs with cancer cells, assessing cytotoxicity, and determining the efficacy of the drug conjugate [106].

2D Cell Lines: In vitro systems are typically the first checkpoint for ADCs. Common BC cell lines such as MDA-MB-231 (a triple-negative subtype), HER2-positive SK-BR-3 [107], and parental human cancer cell lines (BT-474) are foundational in early screening to test antigen expression and cytotoxicity [108]. These lines provide a controlled environment to validate the specificity of ADCs for targeting receptors such as HER2, CEACAM6, or TROP2 [41]. For instance, HER2-positive BC cell lines are frequently used to test HER2-targeted ADCs like trastuzumab emtansine and trastuzumab deruxtecan [109]. These models help researchers determine how well the drug binds, enters the cell, and performs its cytotoxic duties. However, while 2D monolayer cultures are simple and scalable, they fall short of mimicking the tumor’s natural environment.

3D Culture Systems: This is where 3D culture systems become crucial. Tumor spheroids and organoids, often derived from patient samples, more accurately replicate the complex structure and physiology of real tumors than flat cultures. These 3D models preserve the interactions between cancer and stromal cells, providing more precise predictions of drug penetration and therapeutic response [110]. Co-culture spheroids involving JIMT-1 and fibroblasts have demonstrated a strong correlation with in vivo results, underlining their predictive value [107].

In vivo models are equally vital, offering insights into whole-organism pharmacokinetics and toxicity. In vivo experimental frameworks include Cell Line-Derived Xenografts (CDXs) [111], Circulating Tumor Cell-Derived Xenografts (mCTC models) [101], Patient-Derived Xenografts (PDXs) [111,112,113], and Genetically Engineered Mouse Models (GEMMs) [112,113].

Cell Line-Derived Xenografts (CDXs): These are a go-to choice for initial in vivo studies [111]. For example, ADCs specifically directed towards CEACAM6 have demonstrated significant antitumor efficacy in CDX models employing HER2-positive and triple-negative cellular lines [114,115].

Patient-Derived Xenografts (PDXs): PDXs have truly elevated the standard in preclinical cancer research by transplanting actual tumor fragments from patients into immunocompromised mice. Unlike traditional models, PDXs preserve the genetic diversity, molecular features, and receptor expression patterns of the original tumor, making them far more reflective of what happens in real patients [111]. This fidelity makes PDXs a preferred model for testing advanced therapies, such as ADCs, including sacituzumab govitecan [112,113]. Recent innovations have made these models even more practical and scalable—for instance, new protocols now allow for anesthesia-free orthotopic implantation of PDXs directly into the mammary fat pad of NOD scid gamma (NSG) mice. This not only improves animal welfare but also enhances the biological relevance of the model by simulating natural tumor progression more accurately [111]. Altogether, PDXs offer a powerful and increasingly accessible bridge between the laboratory and clinical reality.

Circulating Tumor Cell-Derived Xenografts (mCTC models): These models are gaining momentum, especially for studying metastasis. Derived from MDA-MB-231 xenografts, these cells exhibit enhanced invasive behavior and have helped identify biomarkers, such as SPARC, as potential ADC targets [116].

Genetically Engineered Mouse Models (GEMMs): In conclusion, GEMMs offer a unique benefit due to their complete operational immune system. Though technically demanding, GEMMs are invaluable for evaluating immune interactions and tracking tumor evolution in a native setting [114].

### 6.2. Biomarker Identification and Validation

Biomarkers are at the heart of successful ADC development, guiding both patient selection and anticipating treatment outcomes. Among these, the expression of HER2 stands as the most thoroughly validated and clinically substantiated biomarker, particularly within the framework of HER2-positive breast carcinoma [117]. As elucidated by Bardia, Hurvitz [35], HER2 serves as a fundamental component in the methodologies employed for ADC targeting, thereby laying the groundwork for tailored, precision-driven therapeutic strategies within this specific patient cohort. It underscores ongoing initiatives aimed at refining patient stratification based on HER2 expression levels, particularly concerning the HER2-low and HER2-negative subtypes [35].

Additionally, emerging biomarkers such as tumor-infiltrating lymphocytes and neutrophil-to-lymphocyte ratios are being explored to enhance the predictive accuracy of ADCs in combination with immunotherapies [117]. These emerging biomarkers can be summarized in Table 2, which include TROP2 [112,118,119,120], HER3 [121,122], LIV-1 [122,123,124], Claudin-18.2 [125], B7-H3 [117], CEACAM5 [126], MUC1 [127], and ROR1 [112,127].

The validation of these biomarkers in preclinical settings is essential to ensure their reliability and applicability in clinical trials. This involves rigorous testing in both in vitro and in vivo models to establish a correlation between biomarker expression and ADC efficacy [128].

### 6.3. Translational Challenges and Bridging the Gap

One of the significant challenges in ADC development is translating preclinical findings into human outcomes. Preclinical models often fail to fully replicate the complexity of human tumors, leading to discrepancies in efficacy and toxicity predictions. For example, the heterogeneity of antigen expression in human tumors can result in variable responses to ADCs, which is not always captured in preclinical models [129]. Additionally, the off-target effects and potential toxicities observed in animal models may not accurately predict human responses, and the optimization of ADC components, such as the antibody, linker, and payload, is also crucial [113]. Table 2 represents the emerging ADCS cancer biomarkers.

Due to these challenges, translational studies are essential for bridging the gap between preclinical findings and clinical applications. These studies involve the integration of preclinical data with clinical trial design to optimize dosing regimens, patient selection, and combination therapies [122,130]. Moreover, translational studies facilitate the identification of novel targets and the development of next-generation ADCs with improved efficacy and safety profiles. This iterative process is essential for advancing ADCs from the laboratory to the clinic, ultimately enhancing patient outcomes in BC treatment [112].

**Table 2:** Most common emerging ADCS cancer biomarkers.

Biomarker	Relevance	Current Application	Drug Targeted	References
TROP2	Trophoblast cell surface antigen 2, overexpressed in various solid tumors, including BC, is effective in triple-negative and hormone receptor-positive, HER2-negative BC	Targeted by ADCs like Sacituzumab Govitecan, approved for metastatic BC	Sacituzumab Govitecan, Datopotamab Deruxtecan	[112,118,119,120]
HER3	Human epidermal growth factor receptor 3, which is overexpressed in various cancers, including BC, and associated with resistance to HER2-targeted therapies	Under investigation in clinical trials for ADCs targeting HER3	Patritumab deruxtecan (U3-1402)	[121,122]
LIV-1	Zinc transporter LIV-1, overexpressed in BC, particularly in triple-negative BC (TNBC)	Targeted by ADCs in clinical trials, showing promise in TNBC	Ladiratuzumab vedotin	[122,123,124]
Claudin-18.2	A tight junction protein, highly selective expression in tumors, limited in normal tissues	Targeted in gastric and BCs; potential for ADC development	Zolbetuximab (IMAB362)	[125]
B7-H3	An immune checkpoint molecule overexpressed in various cancers, including BC	Targeted by ADCs in clinical trials, with potential for combination with immunotherapy	Enoblituzumab	[117]
ROR1	Receptor tyrosine kinase-like orphan receptor 1 is expressed in various cancers, including BC	Under investigation for ADC targeting; potential in solid tumors	VLS-101 (in development)	[27,112]
MUC1	Mucin protein overexpressed in BC associated with poor prognosis	Targeted by ADCs in clinical trials, showing potential in various subtypes of BC	oportuzumab monatox	[127]
CEACAM5	Overexpressed in several epithelial tumors, including BC, with limited expression in normal tissues	Targeted by ADCs in preclinical and clinical studies	SAR408701	[126]
SPARC	Secreted protein acidic and rich in cysteine, involved in tumor progression and metastasis	Potential targets for ADCs, though specific drugs are still in development	Not specified	[112,127]

B7-H3, B7 homolog 3; BC, breast cancer; CEACAM5, Carcinoembryonic antigen-related cell adhesion molecule 5; Claudin-18.2, Claudin 18 isoform 2; HER3, Human epidermal growth factor receptor 3; LIV-1, Zinc transporter LIV-1; MUC1, Mucin 1; ROR1, Receptor tyrosine kinase-like orphan receptor 1; SPARC, Secreted protein acidic and rich in cysteine; TROP2, Trophoblast cell-surface antigen 2.

## 7. Immunogenicity of ADCs in BC

ADCs have transformed BC therapy, but their immunogenicity remains a critical challenge. Immunogenicity can alter pharmacokinetics (PK), reduce efficacy, and trigger adverse events, necessitating a nuanced understanding of its drivers, detection methods, and mitigation strategies.

### 7.1. Factors Influencing ADC Immunogenicity

**Figure 2:**
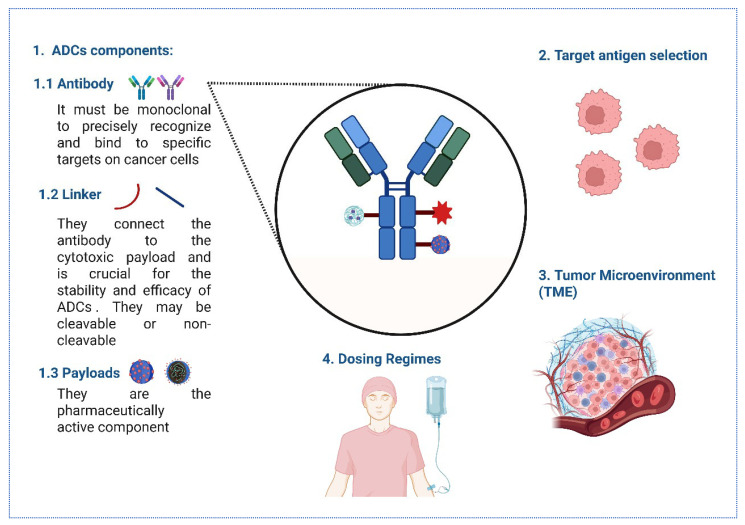
The key considerations for optimizing ADC immunogenicity. (1) ADCs consist of monoclonal antibodies targeting specific cancer cell antigens, linkers (cleavable or non-cleavable) connecting to cytotoxic payloads, and active pharmaceutical payloads. (2) Target antigen selection focuses on high tumor expression and low healthy tissue distribution for precision. (3) The TME impacts ADC penetration and efficacy, depicted with cellular complexity. (4) Dosing regimens, illustrated by intravenous administration, balance therapeutic benefits and toxicity, ensuring effective cancer cell targeting while minimizing off-target effects.

The immunogenicity of ADCs is influenced by their components, including the monoclonal antibody, target antigen selection, linker, and cytotoxic payload. The choice of these components can affect the immune response, with humanized or fully human antibodies generally reducing immunogenicity compared to murine antibodies [131] (Figure 2).

Antibodies used in ADCs need to be monoclonal to precisely recognize and bind to specific targets on cancer cells. They should remain active in the bloodstream for a prolonged period, trigger minimal immune reactions, avoid unintended interactions with healthy tissues, be efficiently absorbed into the target cells, and be capable of killing those cells either directly or by activating the body’s immune defenses [129,132,133]. Human antibodies, or immunoglobulins (Igs), are grouped into five main types—IgM, IgG, IgA, IgE, and IgD—based on the structure of their heavy chain regions. Among all Ig classes, IgG is the most abundant in the bloodstream. It can be further broken down into four subtypes—IgG1, IgG2, IgG3, and IgG4—each with slightly different amino acid structures that influence their function [134]. Full-length IgG antibodies, particularly the IgG1 subclass, are commonly used due to their favorable properties of high specificity and affinity and also due to their long serum half-life [135]. Therefore, the choice of monoclonal antibody is crucial, as it determines the specificity and affinity of the target antigen, thereby influencing the immunogenicity of the ADC [136].

Linker Chemistry: The chemical nature of the linker can also impact immunogenicity [120]. The linker connects the antibody to the cytotoxic payload and is crucial for the stability and efficacy of ADCs [134]. Linkers in ADCs are designed to be stable [134], and they maintain the cancer-killing drug’s attachment during circulation, releasing it only once the ADC reaches the tumor, thereby ensuring precise and effective treatment [137]. Linkers in ADCs fall into two main categories—cleavable and non-cleavable—based on how they release the payload once the ADC is inside the target cell [95]. Cleavable linkers are sensitive to conditions within the target cell, such as acidic pH or specific enzymes, allowing for payload release.

In contrast, non-cleavable linkers rely on the complete degradation of the antibody within the lysosome to release the active payload [134]. Because they are chemically synthesized, these linkers tend to be less toxic and remain in the bloodstream for longer compared to cleavable ones, making them a safer and more durable option [138,139]. A stable linker minimizes premature drug release, which can reduce immunogenic responses [136].

Payload Characteristics: The cytotoxic payload itself can be immunogenic, particularly if it is a novel or foreign compound to the human immune system [113,129]. Innovative payloads, such as immune-stimulating agents and radionuclides, are being explored to enhance specificity and efficacy, but they also pose challenges related to immune activation and potential adverse effects [140]. Common classes of payloads include microtubule inhibitors such as monomethyl auristatin E (MMAE) and maytansinoids, which disrupt cell division [141]; DNA-Damaging Agents such as calicheamicin and pyrrolobenzodiazepines, which lead to DNA strand breaks [134]; and topoisomerase inhibitors like SN-38, which can interfere with DNA replication [139,142].

Target antigen selection: Selecting appropriate target antigens is crucial for minimizing off-target effects and enhancing the specificity of ADCs. Bispecific antibodies, which can target two different antigens, are being investigated to improve targeting precision and reduce immunogenicity risks [143].

The TME: Stromal cells, such as cancer-associated fibroblasts (CAFs), secrete TGF-β, which downregulates target antigens (e.g., HER2) and promotes ADC degradation, thereby fostering antigenic variability and immune evasion [144,145].

Dosing Regimens: Subtherapeutic doses may induce tolerance, while high doses can overwhelm immune surveillance, thereby increasing the incidence of anti-drug antibodies (ADAs) [146].

Besides these factors, ADC immunogenicity can also be influenced by patient-specific factors, such as genetic polymorphisms (e.g., HLA haplotypes), prior exposure to biologic therapies, and immunosuppressed states (e.g., advanced metastatic disease), which heighten immune recognition [147].

Finally, manufacturing impurities—such as protein aggregates, endotoxins, or residual host cell proteins—can function as adjuvants, enhancing the likelihood of an immune response [148]. Stringent quality control and purification steps are thus critical not just for drug efficacy but for immunological safety.

### 7.2. Methods for Assessing ADAs

The detection and analysis of ADAs are crucial in evaluating the immunogenicity of biopharmaceuticals, which can affect drug efficacy and patient safety [149]. The detection methods may include immunoassays, advanced analytical techniques, or generic and emerging assays.

Immunoassays: Electrochemiluminescence (ECL) Immunoassay: This method is used for detecting ADAs in human plasma and serum. It involves capturing ADAs with a biotinylated drug and detecting them with a ruthenylated drug, providing high sensitivity and specificity. For instance, the ECL immunoassay was successfully applied to detect ADAs against the ADC PYX-201 and the monoclonal antibody PYX-106, demonstrating its utility in clinical studies [150,151]. Enzyme-linked immunosorbent assays (ELISAs) and radioimmunoassays are commonly used to detect ADAs. These methods are sensitive and can quantify the presence of antibodies against ADCs [85]. ELISA can be performed in a bridging format, which is effective for screening and confirming ADAs. It is often used in conjunction with other platforms, such as the Meso Scale Discovery system, to enhance detection capabilities [152].

Advanced Analytical Techniques: Immunocapture-Liquid Chromatography/Mass Spectrometry (LC-MS): This emerging technique enables the isotyping and semi-quantification of ADAs. It combines immunocapture with liquid chromatography-tandem mass spectrometry (LC-MS) to provide detailed information on ADA characteristics, offering a robust alternative to traditional immunoassays [153]. Surface Plasmon Resonance (SPR): SPR is a label-free technique that enables real-time monitoring of ADA interactions with ADCs, eliminating the need for labeling [131]. It provides kinetic data on ADA binding, which is valuable for understanding the immunogenicity of biopharmaceuticals [154].

Generic and Emerging Assays: Generic ADA Assays: These assays do not require drug-specific reagents, making them suitable for non-clinical studies. They help reduce assay development time and are useful for initial ADA screening [152]. Neutralizing Antibody Assays: These assays determine the functional impact of ADAs by assessing their ability to inhibit ADC activity, providing a more comprehensive understanding of ADA effects [155]. Homogeneous Mobility Shift Assays: These assays are based on changes in the mobility of ADA–drug complexes during electrophoresis, providing a label-free method for ADA detection [154].

### 7.3. Impact of ADAs on ADC Pharmacokinetics, Efficacy, and Safety

ADAs can significantly influence the pharmacokinetics of ADCs by increasing drug clearance and reducing systemic exposure, which can lead to suboptimal therapeutic levels and reduced efficacy [156]. For instance, in the case of adalimumab, a high incidence of ADA formation was associated with increased drug clearance and disease relapse [111]. The pharmacokinetic profile of ADCs is also affected by the stability of the linker and the nature of the payload, which can be influenced by ADA interactions [157]. The presence of ADAs can neutralize ADCs, diminishing their therapeutic effects and potentially leading to treatment failure [158]. This can result in a loss of response to treatment, as seen with adalimumab, where ADA levels correlate with disease relapse [111].

Safety concerns arise from the potential for ADAs to trigger immune-mediated adverse reactions, which necessitates careful monitoring and management strategies [159,160]. ADAs may also contribute to adverse effects by forming immune complexes that can trigger hypersensitivity reactions or other immune-mediated toxicities [65]. In clinical trials, the efficacy and safety profiles of ADCs, such as rituximab and bevacizumab biosimilars, were shown to be comparable to those of their reference products, despite the presence of ADAs, indicating that biosimilars can maintain efficacy and safety even in the presence of immunogenic responses [160,161].

### 7.4. Strategies to Mitigate Immunogenicity

The immunogenicity of ADCs poses significant challenges, potentially affecting their pharmacokinetics, efficacy, and safety. Various strategies have been developed to mitigate the immunogenicity of ADCs, with a focus on the design and engineering of their components. These strategies aim to enhance the therapeutic index of ADCs while minimizing adverse immune responses [63]. Strategies to mitigate immunogenicity can be summarized as follows:

Humanization of Antibodies: The development of humanized or fully human antibodies can significantly reduce immunogenicity, as these are less likely to be recognized as foreign by the immune system [113], and the immune response is minimized compared to murine or chimeric antibodies. Therefore, protein engineering efforts have focused on optimizing the antibody’s binding affinity and specificity to target antigens [109,143], as modifying the mAb component to reduce immunogenic epitopes can help decrease the immune response [162]. Optimizing Linker and Payload: Selecting non-immunogenic linkers and payloads or modifying them to reduce immunogenic potential can help mitigate immune responses [122].

Conjugation and Combination Techniques: Employing site-specific conjugation methods can produce more homogeneous ADCs with consistent drug-to-antibody ratios, which may reduce immunogenicity and improve therapeutic outcomes [163,164]. Also, using ADCs in combination with other therapies, such as immune checkpoint inhibitors, may help overcome resistance and improve efficacy [165]. Immune Tolerance Induction: Pre-treatment with immunosuppressive agents or co-administration with immune modulators may help induce tolerance to ADCs, reducing ADA formation [129]. Personalized Medicine Approaches: Tailoring ADC treatments based on individual patient profiles and molecular markers can optimize efficacy and reduce immunogenic responses [165].

## 8. Future Directions and Perspectives in ADC Development

### 8.1. Artificial Intelligence and Machine Learning in ADC Design

**Figure 3:**
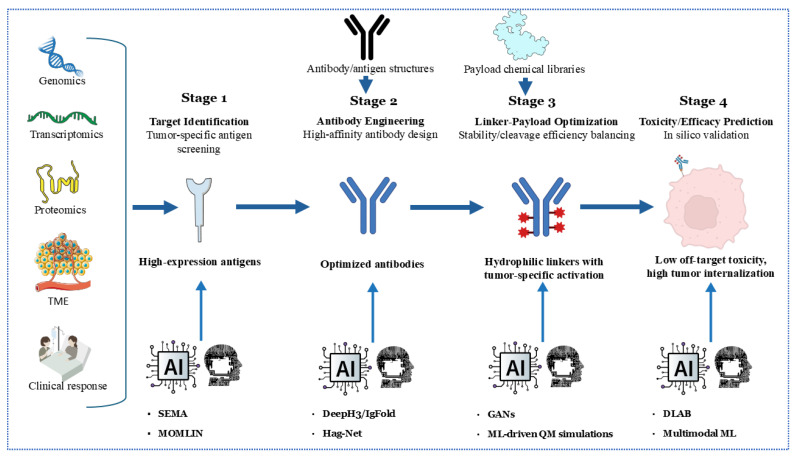
An AI/ML-accelerated development pipeline for antibody–drug conjugates (ADCs) in four stages. Stage 1 uses genomics, transcriptomics, proteomics, and TME analysis with AI tools (SEMA, MOMLIN) to screen tumor-specific, high-expression antigens. Stage 2 employs AI (DeepPhyl, HagNet) for high-affinity antibody engineering and optimization. Stage 3 leverages AI (GANs, ML-driven QM simulations) to design hydrophilic linkers with tumor-specific activation, balancing stability, cleavage, and efficacy. Stage 4 utilizes AI (DLAB, Multimodal ML) to predict toxicity and efficacy in silico, minimizing off-target effects and enhancing tumor internalization, guiding clinical response optimization.

The integration of artificial intelligence (AI) and machine learning (ML) is revolutionizing the development of ADCs across multiple fronts. For target identification, AI algorithms analyze multi-omics datasets (including genomics, proteomics, and transcriptomics) to pinpoint tumor-specific antigens with optimal expression profiles, thereby minimizing on-target/off-tumor toxicity. Tools like SEMA utilize transfer learning to predict B-cell epitopes, facilitating the rapid screening of antigen candidates for ADC targeting [166]. In antibody engineering, deep learning models such as DeepH3 and IgFold predict antibody structures with near-experimental accuracy (CDR-H3 RMSD: 2.2–3.3 Å), facilitating the design of high-affinity antibodies against complex targets [167] (Figure 3).

Recent advancements in multimodal machine learning (ML) integration, such as the MOMLIN framework, demonstrate how AI can correlate multi-omics network biomarkers (e.g., ER-negative status with HMCN1-COL5A1 mutations and CD8+ T-cell infiltration) to predict drug response with AUCs greater than 0.98, outperforming traditional methods by 10–15% [168]. This approach identifies context-specific biomarkers, such as interactions between FLT3 signaling pathways and antimicrobial peptide responses, which could refine ADC target selection and payload optimization.

Linker-payload optimization benefits from ML-driven quantum mechanical simulations, predicting serum stability and payload release kinetics. For instance, generative adversarial networks (GANs) screen linker libraries to balance hydrophilicity and enzymatic cleavage efficiency, reducing systemic toxicity [169]. Toxicity and efficacy prediction is enhanced by graph neural networks (GNNs) like DLAB, which model ADC–antigen binding dynamics to forecast internalization efficiency and off-target effects [170]. A recent work by Kang et al. demonstrates that sequence-based Hag-Net GNNs predict ADC affinity maturation pathways with AUCs greater than 0.95, enabling in silico optimization before synthesis [171]. Future efforts must address algorithm generalizability using standardized training datasets and explainable AI frameworks to validate predictions across diverse tumor contexts.

### 8.2. Personalized ADC Therapy

Personalization of ADCs hinges on tumor molecular subtyping and dynamic biomarker monitoring. “HER2-low” breast cancers (IHC 1+/2+ with negative FISH) exemplify this paradigm, where ADCs like trastuzumab deruxtecan (T-DXd) demonstrate efficacy in traditionally HER2-negative populations [26]. AI-driven multi-omics integration—combining SNP arrays, RNA-seq, and mass cytometry—can identify patient subgroups with aberrant endocytic machinery (e.g., RAB5A overexpression) predictive of ADC internalization.

The MOMLIN framework highlights the potential of integrating clinical, genomic, and transcriptomic data to stratify patients by treatment response. For example, ER-negative tumors with HMCN1 mutations and elevated CSF3R expression exhibit enhanced immune infiltration (CD8+ T-cells), suggesting these patients may benefit from immunostimulatory ADCs [168]. Conversely, resistance-associated biomarkers, such as TP53 mutations and HLA-E upregulation, could guide the development of combinatorial therapies to overcome ADC resistance.

Longitudinal liquid biopsies further refine personalization. Circulating tumor DNA (ctDNA) detects emergent resistance mutations (e.g., HER2 extracellular domain truncations), enabling real-time ADC switching or the use of combinatorial regimens. Clinical trials, such as DAISY (NCT04132960), are validating the use of ctDNA-guided T-DXd sequencing in HER2-ultralow metastatic breast cancer, where AI algorithms correlate ctDNA dynamics with ADC response [172]. Future frameworks will require digital twin models simulating ADC pharmacokinetics at single-cell resolution, informed by patient-specific organoid screens.

### 8.3. Advancements in Imaging and Diagnostics

Companion diagnostics (CDx) for ADCs demand quantitative precision beyond conventional IHC. AI-powered digital pathology platforms now achieve 97% sensitivity and 82% specificity in HER2-low classification, reducing inter-observer variability from 30% to <5% [173]. These systems utilize convolutional neural networks (CNNs) to analyze whole-slide images (WSIs), segmenting tumor regions and quantifying membrane staining intensity and spatial heterogeneity—critical for ADC target engagement.

Emerging deep learning (DL) models, such as those applied in the TransNEO cohort, demonstrate how histopathological features (e.g., tumor-infiltrating lymphocytes [TILs] and stromal composition) can predict response to neoadjuvant therapy [168]. For instance, automated TIL quantification via platforms like QuPath correlates with pathological complete response (pCR), offering a cost-effective alternative to genomic assays for ADC patient stratification.

Emerging molecular imaging techniques enable in vivo ADC tracking. ^89^Zr-labeled ADCs paired with PET-MRI visualize intratumoral distribution, revealing “cold” regions with poor drug penetration. Novel activatable fluorescent linkers enable real-time illumination of payload release, correlating with early responses (e.g., in sacituzumab govitecan-treated TNBC). AI algorithms integrate imaging data with transcriptomics to predict ADC resistance, such as lysosomal protease downregulation, as detected via radiomics features. Future CDx will integrate spatial transcriptomics to map antigen accessibility within the tumor stroma, identifying “ADC-eligible” niches that are masked by bulk analysis.

### 8.4. Overcoming Resistance Through Novel Strategies

ADC resistance arises from target-dependent (antigen loss or modulation) and target-independent (efflux pumps, impaired lysosomal function) mechanisms. To counteract antigen escape, bispecific ADCs (e.g., zanidatamab zovodotin) simultaneously engage HER2 and EGFR, thereby increasing tumor coverage and efficacy in heterogeneous lesions [174]. For efflux-mediated resistance, P-glycoprotein inhibitors (tariquidar) restore the cytotoxicity of the payload in MDR1+ tumors.

Recent multi-omics analyses reveal that resistance is often driven by multimodal interactions, such as TP53 mutations combined with T-cell exclusion signatures [168]. AI models identify these networks, enabling the design of ADCs with payloads targeting resistance pathways (e.g., STING agonists for immune-excluded tumors or BRD4 degraders for TP53-mutant clones).

Resistance-pathway-targeting ADCs represents a frontier. ADCs carrying BRD4 degraders (e.g., GSKBRD4) silence oncogenic transcripts that upregulate survival signals, while STING agonist payloads reverse immune-excluded microenvironments. Sequential therapies also show promise: priming with EGFR inhibitors enhances HER3 ADC uptake by preventing compensatory HER3 upregulation in lung cancer models [175]. Machine learning identifies optimal sequences by modeling tumor clonal dynamics under ADC pressure.

### 8.5. Expanding the Therapeutic Window

Improving ADC safety requires precision targeting and payload engineering. Tumor-activatable linkers (e.g., glucuronidase-cleavable SG3249) minimize premature payload release, reducing neutropenia and interstitial lung disease [176]. Site-specific conjugation ensures homogeneous drug–antibody ratios, thereby avoiding DAR ≥ 8 aggregates that can trigger hepatotoxicity.

AI-driven biomarker discovery, as exemplified by MOMLIN, underscores the significance of stromal–immune interactions in modulating toxicity. For example, tumors with high collagen-dominated stroma exhibit reduced ADC penetration, while lymphocyte-rich microenvironments correlate with both efficacy and lower off-target toxicity [168]. These insights can inform linker design to capitalize on tumor-specific stromal protease activity.

Novel payload classes further widen the window. Immunostimulatory ADCs (iADCs) deliver TLR7/8 agonists to repolarize tumor-associated macrophages, thereby synergizing with checkpoint inhibitors without inducing additional cytotoxicity. Protein degraders (PROTAC-ADCs) achieve efficacy at sub-nanomolar concentrations, lowering systemic exposure. Moreover, computational models can generate synthetic antibody libraries, avoiding aggregation-prone regions and thus enabling the engineering of thermostable variants and mitigating immunogenicity [177]. Future ADCs may exploit tumor microenvironment (TME)-responsive chemistries, such as pH-sensitive duocarmycins activated only in acidic niches.

## 9. Conclusions

The landscape of BC therapy has been profoundly reshaped by antibody–drug conjugates, marking a pivotal shift towards more targeted and effective treatments. This review has underscored the remarkable progress in ADC design, from evolving antibody targets and advanced linker technologies to novel cytotoxic payloads and multi-modal combination strategies. However, the journey towards optimizing ADCs is ongoing, fraught with complexities such as tumor heterogeneity, drug resistance mechanisms, and challenges in predicting patient response and managing toxicity. Future directions are centered on harnessing cutting-edge technologies, such as artificial intelligence, for personalized ADC therapy, developing advanced imaging techniques for precise diagnostics, and devising innovative strategies to circumvent resistance. Ultimately, continuous research and collaborative efforts will be paramount in translating these advancements into superior clinical outcomes, expanding the therapeutic window, and ensuring that ADCs fulfill their immense promise of providing more effective and safer treatment options for BC patients.

## Data Availability

All data generated are presented in the current MS.

## Abbreviations

The following abbreviations are used in this manuscript:

ADC	Antibody–Drug Conjugate
ADCC	Antibody-Dependent Cellular Cytotoxicity
ADCP	Antibody-Dependent Cellular Phagocytosis1
ADAs	Anti-Drug Antibodies
AI	Artificial Intelligence
CDX	Cell Line-Derived Xenografts
CDx	Companion Diagnostics
CDC	Complement-Dependent Cytotoxicity
CDK	Cyclin-Dependent Kinase
CNNs	Convolutional Neural Networks
ctDNA	Circulating Tumor DNA
DAR	Drug-to-Antibody Ratio
DDR1	Discoidin Domain Receptor 1
DL	Deep Learning
ECL	Electrochemiluminescence
EGFR	Epidermal Growth Factor Receptor
ELISAs	Enzyme-Linked Immunosorbent Assays
EpCAM	Epithelial Cell Adhesion Molecule
ER	Estrogen Receptor
FcRn	Neonatal Fc Receptor
FISH	Fluorescence In Situ Hybridization
GANs	Generative Adversarial Networks
GEMMs	Genetically Engineered Mouse Models
GNNs	Graph Neural Networks
HER2	Human Epidermal Growth Factor Receptor 2
HER3	Human Epidermal Growth Factor Receptor 3
IHC	Immunohistochemistry
iADCs	Immunostimulatory Antibody–Drug Conjugates
Igs	Immunoglobulins
LC-MS	Liquid Chromatography/Mass Spectrometry
LGR5	Leucine-Rich Repeat-Containing G-Protein Coupled Receptor 5
mAb	Monoclonal Antibody
mCTCs	Circulating Tumor Cell-Derived Xenografts
mFISHseq	Multiplexed Fluorescence In Situ Hybridization Sequencing
ML	Machine Learning
MMAE	Monomethyl Auristatin E
MOMLIN	Multi-Omics ML Integration
PARP	Poly ADP-Ribose Polymerase
pCR	Pathological Complete Response
PDX	Patient-Derived Xenografts
PK	Pharmacokinetics
PROTAC	Proteolysis Targeting Chimeras
QSP	Quantitative Systems Pharmacology
RMSD	Root Mean Square Deviation
scFVs	Single-Chain Variable Fragments
SG	Sacituzumab Govitecan
SNP	Single Nucleotide Polymorphism
SPARC	Secreted Protein Acidic and Rich in Cysteine
SPR	Surface Plasmon Resonance
T-DM1	Trastuzumab Emtansine
T-DXd	Trastuzumab Deruxtecan
TILs	Tumor-Infiltrating Lymphocytes
TLR	Toll-Like Receptor
TME	Tumor Microenvironment
TNBC	Triple-Negative Breast Cancer
TOP1	Topoisomerase I
TROP2	Trophoblast Cell Surface Antigen 2
WSIs	Whole-Slide Images
